# Lymphangioleiomyomatosis Presenting with Perirenal Hemorrhage

**DOI:** 10.5334/jbr-btr.836

**Published:** 2015-09-15

**Authors:** S. Dekeyzer, N. Peters, P. Smeets, P. De Visschere, K. Decaestecker, R. Gosselin

**Affiliations:** 1 Department of Radiology and medical imaging, University Hospital Ghent, Ghent, Belgium; 2 Department of Urology, University Hospital Ghent, Ghent, Belgium

**Keywords:** Lymphangiomyomatosis

## Abstract

Lymphangioleiomyomatosis (LAM) is a rare debilitating disease of unknown etiology, classically described as almost exclusively affecting women of childbearing age. The disease most commonly involves the lungs and is characterized by hamartomatous smooth muscle cell proliferations along blood vessels, airways and lymphatics. Most patients present with pulmonary symptoms, including shortness of breath, recurrent pneumothorax and pleural effusions. Extrapulmonary manifestations of LAM as the initial presentation of the disease are highly unusual. We present the case of a patient in whom LAM was incidentally discovered when the patient presented with retroperitoneal hemorrhage from a ruptured renal angiomyolipoma.

Lymphangioleiomyomatosis (LAM) is a rare multisystem disorder of unknown etiology, primarily affecting women. The disease mainly affects the lung and is characterized histologically by a diffuse proliferation of smooth muscle cells in the walls of the airways, venules and lymphatic vessels. Proliferation of these cells leads to vascular and airway obstruction, cyst formation and a progressive decline in respiratory function. The most frequent extrapulmonary manifestations include retroperitoneal lymphadenopathies, lymphangioleiomyomas, renal angiomyolipomas and chylous ascites. A symptomatic extrapulmonary manifestation of LAM, preceding pulmonary symptoms and/or diagnosis, is extremely rare. In this paper we describe a patient in whom LAM was incidentally discovered when she presented with spontaneous perirenal hemorrhage (Wunderlich syndrome) from a ruptured renal angiomyolipoma.

## Case report

**Figure 1:**
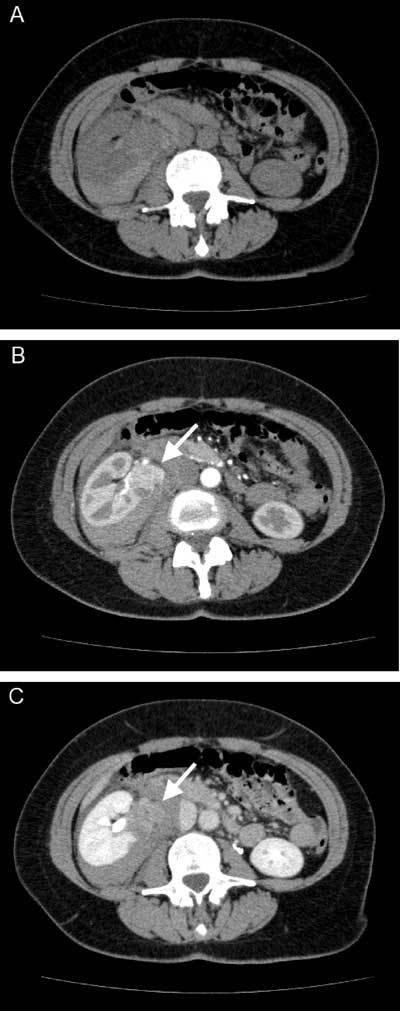
Non-contrast enhanced (A) and arterial (B) and venous (C) phase contrast enhanced CT of the abdomen shows a hemorrhage in the right perirenal space and an exophytic hypervascular mass (*white arrows* in **B** and **C**) with rapid contrast washout extending from the lower pole of the right kidney.

**Figure 2:**
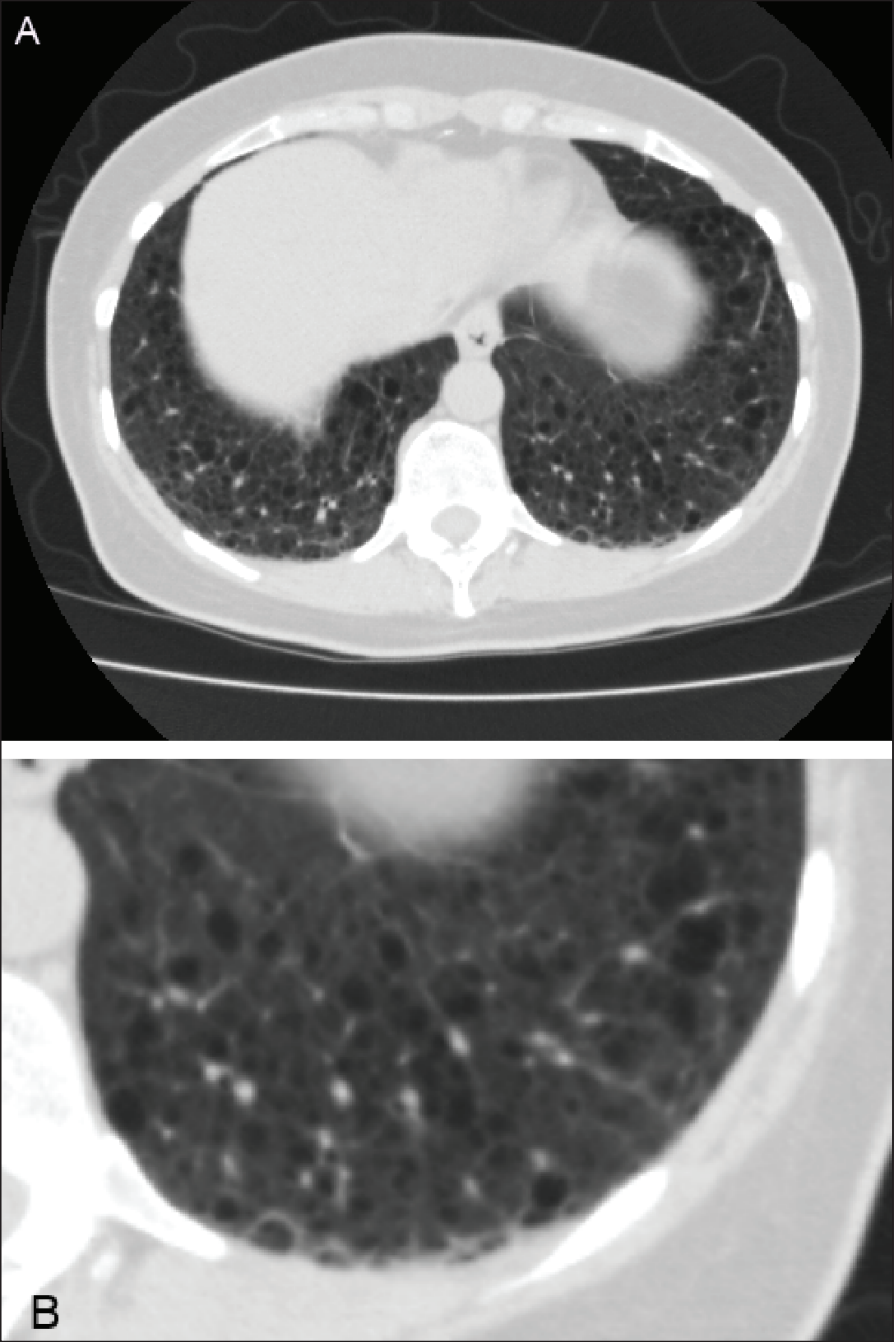
Lung window view of non-contrast enhanced CT abdomen shows multiple thin walled cysts in both costophrenic recesses (A). The cysts can be better appreciated on the magnified view of the base of the left lung (B).

**Figure 3:**
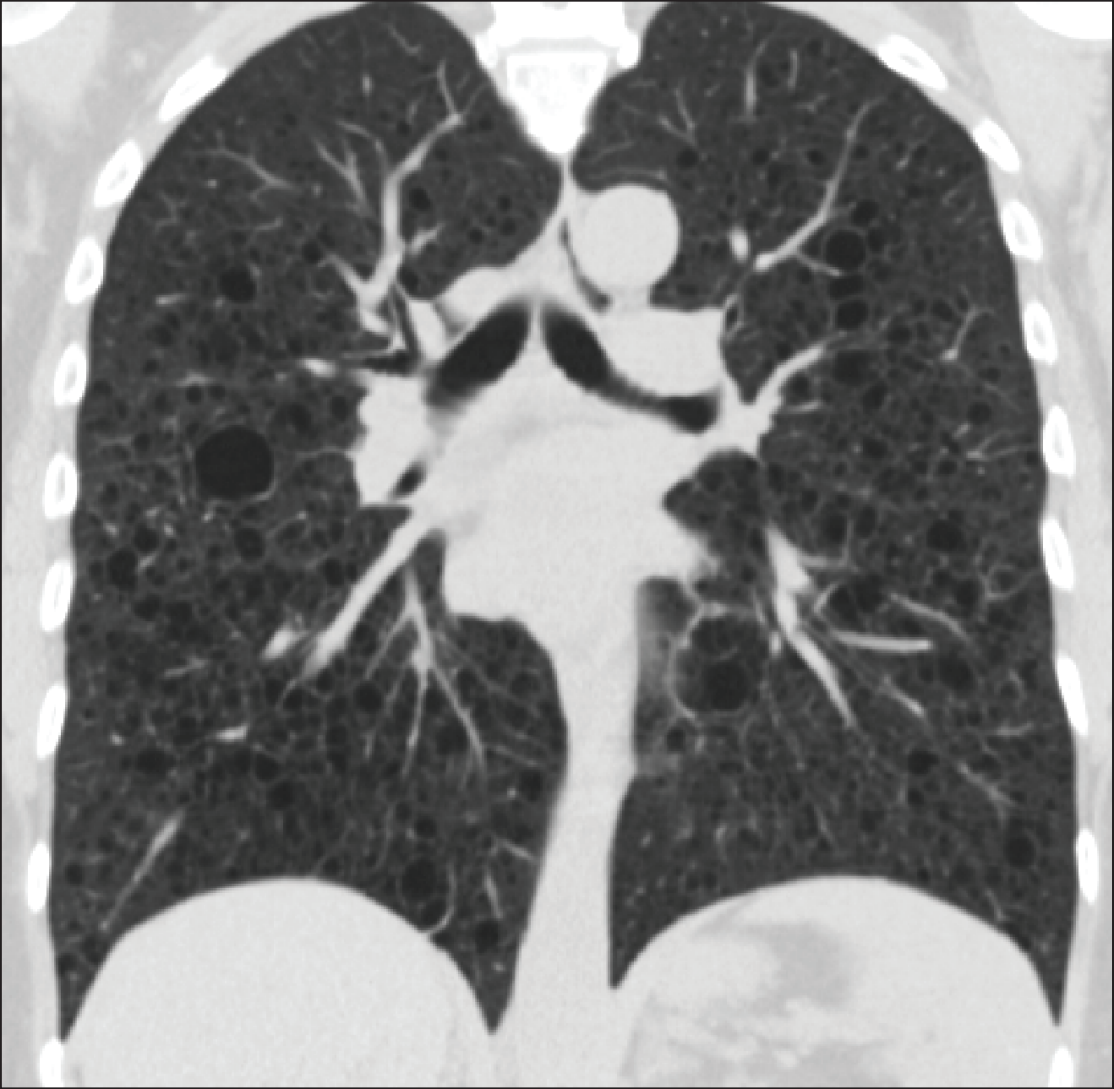
Contrast enhanced CT of the chest shows multiple thin-walled cysts homogenously distributed throughout the lung parenchyma. There are no nodules. Lung apices and bases are equally involved.

A 42-year-old woman presented at the emergency department with acute onset of right flank pain. The patient had an extensive past medical history of endometriosis for which she underwent several surgeries. Contrast enhanced computed tomography (CT) of the abdomen showed right perinephric hemorrhage and an exophytic hypervascular mass arising from the lower pole of the right kidney with a maximum diameter of 5.5 cm (Fig. 1). In the lung bases multiple thin-walled cysts were observed (Fig. 2). Though the tumor did not demonstrate the intratumoral fat density typical for angiomyolipoma, the concomitant presence of cystic lung disease in a premenopausal woman was suggestive of spontaneous retroperitoneal hemorrhage from a renal angiomyolipoma in a patient with lymphangioleiomyomatosis. Based on the imaging characteristics hypernephroma could not be ruled out however. Subsequent CT of the thorax, performed several days later, showed multiple thin-walled cysts of various sizes spread throughout the lung parenchyma. The lung apices and lung bases were equally involved. No nodules were present. Based on these imaging findings a definite radiological diagnosis of LAM was made (Fig. 3). A brain MRI performed to rule out tuberous sclerosis complex (TSC) was normal. The retroperitoneal hemorrhage was treated conservatively. The patient underwent a follow-up CT abdomen two months later. The perirenal blood had completely disappeared. The renal tumor was unchanged in volume and imaging characteristics. Once again no intratumoral fat could be detected. The patient was transferred to an academic center with expertise in interstitial lung diseases and lung transplantation for further follow-up. Because hypernephroma could not be ruled out based on the CT characteristics and because, even if the lesion was to be a benign angiomyolipoma, the patient was prone to rehemorrhage, a right partial nephrectomy was performed. Pathology confirmed the presence of an angiomyolipoma.

## Discussion

Lymphangioleiomyomatosis (LAM) is a rare multisystemic disease of unknown etiology. The disease is classically described as “almost exclusively affecting women of reproductive age” [1]. However, recent studies have demonstrated that LAM occurs in postmenopausal women as well, making some authors suggest that LAM should be defined as a disease of women of childbearing age *and* of middle-aged women [2]. LAM occurs in a sporadic form in patients with no evidence of genetic disease and in about one third of women with tuberous sclerosis complex (TSC) [1,2,3]. The estimated incidence of sporadic LAM is between 2 to 5 cases per 1,000,000 women [2]. This figure is probably an underestimation however, as LAM is often clinically mistaken for asthma or chronic obstructive lung disease. LAM typically follows an insidious clinical course with a variable rate of progression ranging from a few years to over three decades, before eventually culminating in respiratory failure and cor pulmonale [1,2,3].

Although LAM is a multisystemic disease, it mainly affects the lungs. Microscopically, the disease is characterized by hamartomatous proliferations of immature smooth muscle cells (*leiomyo*) surrounding the lymphatics (*lymph*), blood vessels (*angio*) and airways [1,2,3]. The most common pulmonary manifestations of LAM – recurrent spontaneous pneumothorax, chylous pleural effusions and hemoptysis – can all at least in part be explained by the proliferation of smooth muscle cells. Smooth muscle cell proliferation around the bronchioles leads to airway narrowing and obstruction, air trapping, cyst formation and recurrent pneumothorax. Obstruction of pulmonary venules causes vascular congestion and hemoptysis. Lymphatic obstruction leads to chylothorax.

Chest X-ray may be normal early in the course of the disease, but as the disease progresses, diffuse reticular or miliary changes and hyperinflation may develop [4]. High Resolution CT (HRCT) of the chest often shows multiple thin-walled cysts of various shapes and sizes, homogeneously distributed throughout all lung zones. The cysts are characteristically surrounded by normal lung parenchyma. In patients with severe cystic lung disease, the parenchyma can be completely replaced by cysts [4].

The most important radiologic differentials for LAM are centrilobular emphysema, Langerhans cell histiocytosis (LCH) and idiopathic pulmonary fibrosis (IPF). In centrilobular emphysema, the so-called cystic changes are often devoid of distinguishable walls as they represent destroyed alveolar spaces rather than true cysts. Contrary to LAM, where the cysts tend to be uniformly divided over the lung parenchyma, centrilobular emphysema also demonstrates a predilection for the upper lung zones. Upper field preference with relative sparing of the lung bases can also be seen in LCH. Other distinguishing characteristics of LCH are that it includes both nodules and cysts, and that the cysts often have irregular shapes and thicker walls then in LAM. The cystic changes in IPF generally consists of honeycomb abnormalities with a subpleural and basal predominance. Furthermore the honeycomb cysts in IPF are surrounded by abnormal and distorted lung parenchyma, whereas the cysts in LAM have normal intervening lung parenchyma.

Although LAM is primarily a disease of the lungs, extrapulmonary manifestations are present in more than 70% of LAM patients, consisting of enlarged lymph nodes (40%), lymphangioleiomyomas (20%), chylous ascites (10%) and renal angiomyolipomas (AML) (39–93%) [5,6]. The most common of these is renal angiomyolipoma (AML). Renal AML’s are benign tumors of mesenchymal origin. They occur in 93% of patients with TSC associated LAM and in about 30–50% of patients with sporadic LAM [6]. Renal AML’s vary in size from 1 mm to over 20 cm [7]. In patients with TSC associated LAM, the lesions tend to be bilateral, multiple and larger. In patients with sporadic lesion, the lesions are frequently singular and smaller [7].

Microscopically, AML’s are characterized by the presence of adipose tissue, smooth muscle cells and blood vessels. The smooth muscle cells of AML are different from normal smooth muscle cells in that they react (like LAM cells) with monoclonal antibody HMB-45. As HMB-45 immunoreactivity cannot be found in other renal tumors, this antibody is very useful in discriminating AML for other renal masses [7]. The presence of fat aids in the radiological diagnosis of the tumor, as the demonstration of intratumoral fat with negative attenuation values at CT is virtually pathognomonic of AML [5]. However, the absence of fat in a renal lesion should not preclude the diagnosis of an AML in a patient with LAM, as up to 5% of these tumors are lipid-poor [5].

The manifestations of renal AML’s depend on their size. Most renal AML’s are small, asymptomatic and detected incidentally during abdominal ultrasound or CT [7]. Multiple and large masses are more likely to be symptomatic and can present with hematuria, flank pain and retroperitoneal hemorrhage [7]. Retroperitoneal hemorrhage is a serious complication of AML’s and directly related to tumor size. AML’s are vascular tumors, composed of abnormal abundant elastin-poor vascular structures, making them prone to aneurysm and hemorrhage. Generally, the risk of spontaneous retroperitoneal hemorrhage (so-called Wunderlich syndrome) is low when the tumor is less then 4 cm, but increases with tumor size when tumors are more then 4 cm [7]. The hemorrhage is generally confined to the perirenal space.

LAM presenting with abdominal pain is rare and only a limited number of cases can be found in the medical literature. Most cases involve patients presenting with abdominal symptoms caused by large retroperitoneal lymphangioleiomyomas [7,8,9,10]. To our knowledge only one other case has been published of LAM presenting with acute abdominal pain due to a ruptured renal AML [11].

The etiology of sporadic LAM is not yet completely understood. A lot has been learned from the study of TSC as sporadic LAM and TSC probably share a common genetic origin [3,7]. TSC is an inheritable autosomal dominant disorder caused by germline mutations in two tumor suppressor genes: TSC1 on chromosome 9q34 and TSC2 on chromosome 16p13. A second somatic mutation, termed loss of heterozygosity, results in loss of the gene product in that cell. As the TSC-genes are tumor suppressor genes, this allows cellular proliferation and the formation of hamartomas. The TSC2 gene has also been implicated in sporadic LAM, as somatic mutations in the TSC2 gene with loss of heterozygosity are identified in AML’s and pulmonary smooth muscle cells from patients with sporadic LAM. In patients with TSC, germline mutations are present in both tumor and normal tissue. In patients with sporadic LAM however, TSC-2 mutations can be found in the AML and pulmonary LAM cells, but not in the normal tissue. This suggest the possibility of an unusual mechanism of disease: the migration of angiomyolipomatous tissue to lymphatic, abdominal or thoracic organs after arising in a single location. Further research is needed to conform this migratory hypothesis.

Treatment regimens for LAM are primarily based on the notion that the disease typically occurs in women of childbearing age, which suggests that hormonal stimulation plays a role in the disease process. Therefore most therapeutic approaches are aimed at diminishing the effect of estrogen, most commonly by use of intramuscular progesterone [12]. Due the rarity of the disease controlled clinical trials are lacking however and evidence of the use of hormone therapy, even for progesterone, is empiric and weak. In patients with end-stage LAM, lung transplantation, generally of single lungs, has been successfully carried out with survival rates comparable to that of lung transplantations for other indications (i.e. emphysema and pulmonary fibrosis) [4].

## Conclusion

Although LAM mainly involves the lungs, this case illustrates that it is a multisystem disorder that can involve several organs. Extrapulmonary manifestations can be found in about 70% of patients and in rare cases they can even be the first presentation of the disease. Our patient presented with retroperitoneal hemorrhage from a renal AML. There is a clear association between pulmonary LAM and renal AML, and between renal AML’s and spontaneous retroperitoneal hemorrhage. The absence of fat in a renal lesion should not exclude the possibility of AML in a patient with LAM, as no fat can be detected in up to 5% of these tumors.

## Competing Interests

The authors declare that they have no competing interests.
